# Association of TNFSF4 Polymorphisms with Vogt-Koyanagi-Harada and Behcet’s Disease in Han Chinese

**DOI:** 10.1038/srep37257

**Published:** 2016-11-22

**Authors:** Sha Lu, Shengfang Song, Shengping Hou, Hua Li, Peizeng Yang

**Affiliations:** 1 Department of Ophthalmology, Yongchuan Hospital, Chongqing Medical University, Chongqing, P R China; 2 The First Affiliated Hospital of Chongqing Medical University, Chongqing Key Laboratory of Ophthalmology, and Chongqing Eye Institute, Chongqing, P R China

## Abstract

To investigate whether single nucleotide polymorphisms (SNPs) of the Tumor Necrosis Factor Superfamily 4 (TNFSF4) gene are associated with Vogt–Koyanagi–Harada (VKH) and Behcet’s disease (BD) in a Chinese Han population. A two-stage case control study was carried out in 1331 VKH, 938 BD and 1752 healthy controls. Ten TNFSF4 SNPs, including rs1234314, rs1234315, rs2205960, rs704840, rs2795288, rs844654, rs12039904, rs10912580, rs844665, and rs844644, were genotyped using the PCR-restriction fragment length polymorphism method. Genotype and allele frequencies were analyzed between cases and healthy controls using the X^2^ or Fisher’s exact test and p values were corrected for multiple comparisons. We observed a significantly increased frequency of the TT genotype of rs1234315 in BD patients (Pc = 1.44 × 10^−5^, OR = 1.734, 95% CI = 1.398–2.151). The frequency of the TT genotype of rs12039904 was significantly higher in patients with VKH disease as compared to controls (Pc = 4.62 × 10^−5^, OR = 1.959, 95% CI = 1.483–2.588). Analysis of clinical manifestations in VKH disease and BD did not show an association with the TNFSF4 gene polymorphisms. The study suggests that the TNFSF4 gene may be involved in the susceptibility to VKH disease and BD in Han Chinese.

Vogt-Koyanagi-Harada (VKH) disease and Behcet’s disease (BD) are two of the most common uveitis entities in China, manifesting as bilateral panuveitis and can lead to serious visual impairment^1^,^2^,^3^. Although the etiologic pathways or mechanisms of VKH disease and BD remain unclear, many studies have indicated that an autoimmune response following cutaneous injury or a viral infection may trigger an inappropriate and overactive T cell-mediated autoimmune disease, which leads to tissue damage in individuals with a certain genetic background^4^,^5^,^6^. Earlier studies showed that a number of human leukocyte antigen (HLA) and non-HLA genes are associated with VKH disease and BD^7^,^8^,^9^,^10^,^11^,^12^,^13^. However, the susceptibility genes identified thus far do not yet completely explain the genetic pathogenesis of VKH disease and BD.

Recently, several SNPs in a Tumor necrosis factor superfamily-4 (TNFSF4) have been shown to be shared risk factors for several immune-related diseases, including systemic lupus erythematosus (SLE), primary Sjögren’s syndrome (SS) and systemic sclerosis (SS)^14^,^15^,^16^,^17^,^18^,^19^,^20^,^21^,^22^. TNFSF4, also known as OX40L, is expressed on antigen-presenting cells (APC), T cells, B cells, natural killer (NK) cells, macrophages, dendritic cells and vascular endothelial cells^23^. Moreover, TNFSF4 with its unique receptor OX40 as a co-stimulatory signal can activate CD4+ T cells^24^.

Previous studies have shown the important role of TNFSF4 in the development of autoimmune diseases. The association of TNFSF4 polymorphisms with BD and VKH disease has however not been investigated and was therefore the subject of the study presented here. Our results show that polymorphisms of rs1234315 of TNFSF4 are associated with susceptibility to ocular BD, whereas rs12039904 is associated with susceptibility to VKH disease in a Han Chinese population.

## Results

### Clinical features of patients with BD and VKH disease

The demographics and clinical characteristics of the enrolled BD and VKH disease patients in the study are displayed in Tables 1 and 2. The genotype and allele frequencies of the ten SNPs did not deviate from the Hardy-Weinberg equilibrium in the controls. The primers and restriction enzymes used for RFLP analysis of the TSFNF4 gene are shown in Table 3.

### Associations of the TNFSF4 gene polymorphisms with susceptibility to BD

In the first stage of this case-control study, ten SNPs of the TNFSF4 gene were successfully genotyped in 394 BD patients and 606 normal controls. The frequencies of the rs1234315 genotype CC and C allele were significantly lower in patients with BD (Bonferroni Pc^a^ = 1.68 × 10^−4^, OR = 0.484, 95% CI = 0.352–0.664; Pc^a^ = 5.91 × 10^−3^, OR = 0.676, 95% CI = 0.564–0.809; respectively) (Table 4). To confirm this result, another 544 BD patients and another set of 413 normal controls were used to replicate the association between SNP rs1234315 and BD. Increased frequencies of the rs1234315 genotype C allele was observed in BD patients (Pc = 1.07 × 10^−3^, OR = 0.682, 95% CI = 0.568–0.818) as compared to the control group (Table 5). The combined data of the two studies showed a significant association of SNP rs1234315 with BD (Pc^**combined**^ = 1.60 × 10^−6^, OR = 0.688, 95% CI = 0.606–0.780) (Table 5). We could not detect a significant association between the other nine SNPs and BD (Table 4).

### TNFSF4 gene confers susceptibility to VKH disease

Ten SNPs were genotyped in a total of 395 VKH patients and 606 healthy controls in the first-stage study. The frequency of the TT genotype of rs12039904 in VKH patients (Pc^b^ = 4.74 × 10^−3^, OR = 2.494, 95% CI = 1.532–4.060) was significantly higher than that in normal controls (Table 4). Based on this result, we performed a confirmatory study with another set of 936 VKH patients and 1146 normal subjects. The result again demonstrated a significantly higher frequency of the rs12039904 genotype TT in patients with VKH (Pc = 0.034, OR = 1.746, 95% CI = 1.244–2.452) (Table 6). The combined data of the two studies in VKH showed that the TT genotype and T allele of rs12039904 were significantly increased (Pc^**combined**^ = 4.62 × 10^−5^, OR = 1.959, 95% CI = 1.483–2.588; Pc^**combined**^ = 3.48 × 10^−5^, OR = 1.325, 95% CI = 1.183–1.485) as compared to normal controls (Table 6). Genotype and allele frequencies of the other nine SNPs did not reveal a significant difference between the VKH patients and normal controls (Table 4).

## Discussion

In the present study, we show that TNFSF4 gene polymorphisms are associated with both BD and VKH disease, although each disease is associated with a separate locus. The T allele and TT genotype of rs1234315 of TNFSF4 was increased in BD, whereas the T allele and TT genotype of rs12039904 confers risk to VKH disease.

TNFSF4 is mostly expressed on CD4^+^ T cells, where it plays a role in the stimulation of IL-17 production and inhibition of IL-10 production^25^,^26^,^27^. TNFSF4 activates CD4^+^ T cells when bound to its receptor TNFRSF4 as a potent co-stimulatory signal^28^. In addition to CD4+ T cells effects, the co-stimulatory signal inhibits the formation of Foxp3+ regulatory T cells^29^. Recently, Zhou demonstrated a modification of cytokine production in PBMC in Lupus Nephritis patients after treatment with anti-OX40 monoclonal antibody^30^. Moreover, higher serum TNFSF4 levels have been demonstrated in SLE patients as compared to controls, suggesting a role for this molecule as a disease marker^31^. In addition, OX40 activating antibody prolonged and exacerbated disease in a model of experimental autoimmune uveitis by upregulating IL-7Rα expression in the activated T-cell population^32^. Taken together, these findings suggest alink between a genetic variant in the TNFSF4 gene and autoimmune disease^30^,^31^,^32^,^33^.

VKH disease and BD are both immune mediated diseases resulting from an aberrant reaction following environmental triggers in a genetically predisposed individual^34^,^35^,^36^,^37^. In the past, we have reported various immune response related genes to be involved in the susceptibility to both BD and VKH disease, including CD40, JAK2, STAT4, STAT3^38^, ^39^, ^40^, ^41^. To date, the genetic background of BD and VKH disease is however not yet completely clear. Recent studies showed that certain polymorphisms of TNFSF4 increased the risk of autoimmune diseases, such as for instance SLE, SS and primary Sjögren’s syndrome^14^,^15^,^16^,^17^,^18^,^19^,^20^,^21^,^22^. A polymorphism of rs1234315 was reported to be involved in the susceptibility to SLE in European and Asian (including Chinese Han) patients and also in Sjögren’s syndrome^14^,^15^,^16^,^17^,^18^,^22^. In our study, rs1234315 was observed to be associated with BD, whereby the risk T allele was the minor allele in BD patients, which is similar to the association observed in Chinese SLE patients^16^,^18^. Our finding that the rs12039904 of TNFSF4 is associated with VKH disease is similar to findings in European SLE and SS patients, that reported a significant association with the T allele and the TT genotype of rs12039904^14^,^15^. We were not able to confirm earlier reported associations in BD or VKH diseases with other TNFSF4 polymorphisms, whereby rs1234314, rs2205960 and rs844644 showed an association with SLE and SS, and rs704840, rs2795288, rs844654, rs10912580 and rs844665 showed an association with SLE^13^,^14^,^15^,^16^,^17^,^19^,^20^,^21^. This indicates that the pathogenesis of BD and VKH is different from these other autoimmune mediated diseases. The reason why rs1234315 was associated with BD but not with VKH, and that rs12039904 had a significant association with VKH but not with BD is not yet clear but may be due to the fact that both diseases are caused by different pathogenic pathways each leading to serious intraocular inflammation. BD is thought to be caused by an aberrant inflammatory response against microbial antigens, whereas VKH is a disease mediated by an autoimmune response directed against melanocyte antigens^42^,^43^.

Our study has several limitations. We showed that rs1234315 SNP or rs12039904 of TNFSF may play a role in the susceptibility for BD and VKH but we cannot rule out that other as yet not identified SNPs in the TNFSF4 gene may also be associated with these two uveitis entities. Further studies should be performed to investigate whether TNFSF4 is also involved in the pathogenesis of other uveitis entities. The effect of the polymorphisms on the biological function or expression of TNFSF is not yet known and we can therefore only speculate on the exact mechanism by which rs1234315 of TNFSF4 exerts its role in the pathogenesis of BD as well as how rs12039904 affects VHK development.

In conclusion, this study shows an association between polymorphisms of rs1234315 of TNFSF4 with susceptibility to ocular BD, and rs12039904 with susceptibility to VKH in Chinese Han. These data add to the existing knowledge concerning the complex pathogenesis of BD and VKH disease.

## Materials and Methods

### Study population

All patients and healthy controls were recruited from the Zhongshan Ophthalmic Center (Guangzhou, China) and the First Affiliated Hospital of Chongqing Medical University (Chongqing, China) were enrolled from April 2005 to June 2014 and were all Han Chinese. The investigated patients and healthy controls gave a written informed consent before collection of blood. This study was approved by the local ethics research committee at the Chongqing Medical University and was conducted according to the Declaration of Helsinki Principles.

In the first-stage study of this case-control study, 394 BD patients, 395 VKH patients and 606 healthy controls were enrolled to identify susceptible SNPs (Pc < 0.05) in the TNFSF4 gene. In the second-stage, another 544 BD and 413 healthy individual, and another 936 VKH and 1166 controls were added to replicate the susceptible SNPs found in the first-stage study. The diagnosis of BD was based on the criteria of the International Study Group for BD^44^. VKH disease was diagnosed according to the First International Workshop criteria^45^. Clinical characteristics of BD and VKH patients were summarized in Tables 1 and 2. If the diagnosis was in doubt, the patients were excluded from the study.

### DNA extraction and genotyping

Genomic DNA was extracted from blood samples by using the QIAamp DNA Blood Mini Kit (Qiagen, Valencia, California, USA).The proper primers of rs1234314, rs1234315, rs2205960, rs704840, rs2795288, rs844654, rs12039904, rs10912580, rs844665, and rs844644 for amplifying target DNA sequence by polymerase chain reaction- restriction fragment length polymorphism (PCR-RFLP) are depicted in Table 3. Digestion products were separated on 3–5% agarose gels which were stained with GoldView TM (SBS Genetech Beijing, China) following electrophoresis. Five percent of all samples were randomly selected to check the accuracy of genotyping by the Sangon Biotechnology Company (Shanghai, China).

### Statistical Analysis

The X^2^ test was used to check whether the data conformed to the Hardy-Weinberg (HWE) principle. Statistical analysis was performed using SPSS 17.0 software (SPSS, Inc, Chicago, Illinois, USA). The frequencies of genotypes and alleles were evaluated using a case-control study design and significance was tested using the Pearson X^2^ test. The patterns of linkage disequilibrium of the SNPs of TNFSF4 were compared using Haploview 4.0 (Daly Lab at the Broad Institute, Cambridge, Massachusetts, USA). P values were corrected for multiple comparisons using the Bonferroni correction by multiplying obtained p values with the number of analyses performed. A Bonferroni corrected P values <0.05 was considered to be statistically significant.

## Additional Information

**How to cite this article**: Lu, S. *et al*. Association of TNFSF4 Polymorphisms with Vogt-Koyanagi-Harada and Behcet’s Disease in Han Chinese. *Sci. Rep.* **6**, 37257; doi: 10.1038/srep37257 (2016).

**Publisher’s note:** Springer Nature remains neutral with regard to jurisdictional claims in published maps and institutional affiliations.

## Figures and Tables

**Table 1:** Clinical Features of BD Patients used for the first and second stage study.

Clinical Features	BD Patients in the first stage		BD Patients in the second stage	
	Total (394)	%	Total (534)	%
Age at onset (years ± SD)	33.5 ± 8.2		33.9 ± 9.1	
Male	336	85.3	445	83.3
Female	58	14.7	89	16.7
Uveitis	394	100	534	100
Oral ulcer	376	95.4	515	96.4
Genital ulcer	165	41.9	245	45.9
Hypopyon	110	28.0	127	23.8
Skin lesions	190	48.2	282	52.8
Positive pathergy test	132	33.5	152	28.5
Arthritis	112	28.4	143	26.8

**Table 2:** Clinical Features of VKH Syndrome Patients used for the first and second stage study.

Clinical Features	VKH Patients in the first stage		VKH Patients in the second stage	
	Total (395)	%	Total (936)	%
Age at onset (years ± SD)	40.9 ± 14.2		39.9 ± 14.0	
Male	223	56.5	524	56.0
Female	172	43.5	412	44.0
Headache	170	43.0	379	40.5
Dysacusia	149	37.7	294	31.4
Tinnitus	191	48.4	395	42.2
Vitiligo	73	18.5	149	15.9
Alopecia	153	38.7	330	35.3
Poliosis	149	37.7	321	34.3

**Table 3:** Primers and Restriction Enzymes Used for RFLP Analysis of the TSFNF4 Gene.

SNP	Primers	Tm (°C)	Restriction Enzymes
rs1234314	5′-AACAATGAAATGAAATAAATGAT-3′	55	ScrFI
	5′-TCTCCCTCCTTTCTTTACATATC-3′		
rs1234315	5′-GAACACGTTTTCACAGCTCTTCAC-3′	58	TscAI
	5′-TGAAGATGGAGGACTCTGCTTATGT-3′		
rs2205960	5′-AACCTTGGTCTCCTATAATGGGTACTCT-3′	57	Cfr13I
	5′-GACTTTTTCCCTTTGTCATTTCGG-3′		
rs704840	5′-TGGGTGGATAAGCAAAGGGA-3′	57	Bc1I
	5′-GAGATCTGGGCTCATCTAATCTGA-3′		
rs2795288	5′-ATTGGGGGTAGCACATGGAGTT-3′	60	HpyF3I
	5′-CATGCACAAGCACACAATCTGC-3′		
rs844654	5′-ATTGTGGCTGTTACTCTGTCATGG-3′	60	HinfI
	5′-AACTTCCCTTCCCAAGGCTATG-3′		
rs12039904	5′-CTGACCTCCTTGCTCATAGTTGC-3′	58	Mn1I
	5′-TGGCTTCTGTTGGGTCCTTC-3′		
rs10912580	5′-CAAAGCAAGACCCTGACTCA-3′	60	AccI
	5′-CATCTGTTTTGCCTTAGTAG-3′		
rs844665	5′-CCAAAACTGAACTCACCATGTC5′-3′	60	FokI
	5′-GCCTCACAGACAATGGAAAACA-3′		
rs844644	5′-CCTCCATAGACATTCTCTTTCCAGT5′-3′	57	RsaI
	5′-GCCAAGGCAATATATAAAGTGTG-3′		

**Table 4:** Frequencies of alleles and genotypes of TNFSF4 polymorphisms in BD, VKH patients and controls in first stage study.

SNPs	Genotype/allele	BD n (freq)	VKH n (freq)	Controls n (freq)	P^a^/Pc^a^	OR (95% CI)	P^b^/Pc^b^	OR (95% CI)
rs1234314	CC	113 (0.287)	138 (0.349)	184 (0.304)	0.569/NS	0.922 (0.698–1.219)	0.130/NS	1.232 (0.940–1.613
	CG	189 (0.480)	181 (0.458)	296 (0.488)	0.787/NS	0.966 (0.749–1.245)	0.349/NS	0.886 (0.687–1.142)
	GG	92 (0.234)	76 (0.192)	126 (0.208)	0.338/NS	1.161 (0.856–1.574)	0.550/NS	0.908 (0.660–1.247)
	C	415 (0.527)	457 (0.578)	664 (0.548)	0.353/NS	0.918 (0.767–1.099)	0.177/NS	1.133 (0.945–1.357)
	G	373 (0.473)	333 (0.422)	548 (0.452)	0.353/NS	1.089 (0.910–1.304)	0.177/NS	0.883 (0.737–1.058)
rs1234315	CC	66 (0.168)	122 (0.309)	178 (0.294)	5.60 × 10^−6^/1.68 × 10^−4^	0.484 (0.352–0.664)	0.610/NS	1.075 (0.815–1.416)
	CT	228 (0.579)	173 (0.294)	316 (0.521)	0.076/NS	1.260 (0.976–1.628)	0.010/NS	0.715 (0.554–0.923)
	TT	100 (0.254)	100 (0.253)	112 (0.185)	9.10 × 10^−3^/NS	1.500 (1.105–2.037)	0.010/NS	1.495 (1.101–2.030)
	C	360 (0.457)	417 (0.528)	672 (0.554)	1.97 × 10^−5^/5.91 × 10^−3^	0.676 (0.564–0.809)	0.243/NS	0.898 (0.750–1.075)
	T	428 (0.543)	373 (0.472)	540 (0.446)	1.97 × 10^−5^/5.91 × 10^−3^	1.480 (0.236–1.772)	0.243/NS	1.113 (0.930–1.332)
rs2205960	GG	203 (0.515)	187 (0.473)	327 (0.540)	0.450/NS	0.907 (0.703–1.169)	0.041/NS	0.767 (0.595–0.989)
	GT	173 (0.439)	183 (0.463)	248 (0.409)	0.350/NS	1.130 (0.874–1.460)	0.091/NS	1.246 (0.965–1.609)
	TT	18 (0.046)	25 (0.063)	31 (0.051)	0.695/NS	0.888 (0.490–1.610)	0.414/NS	1.253 (0.728–2.157)
	G	579 (0.735)	557 (0.705)	902 (0.744)	0.637/NS	0.952 (0.776–1.168)	0.054/NS	0.822 (0.673–1.004)
	T	209 (0.265)	233 (0.295)	310 (0.256)	0.637/NS	1.050 (0.856–1.288)	0.054/NS	1.217 (0.996–1.487)
rs704840	GG	59 (0.150)	65 (0.165)	91 (0.150)	0.986/NS	0.997 (0.699–1.422)	0.539/NS	1.115 (0.788–1.577)
	GT	219 (0.556)	187 (0.473)	311 (0.513)	0.187/NS	1.187 (0.920–1.531)	0.219/NS	0.853 (0.662–1.099)
	TT	116 (0.294)	143 (0.362)	204 (0.337)	0.162/NS	0.822 (0.625–1.082)	0.409/NS	1.118 (0.857–1.458)
	G	337 (0.428)	317 (0.401)	493 (0.407)	0.354/NS	1.090 (0.909–1.307)	0.806/NS	0.977 (0.814–1.173)
	T	451 (0.572)	473 (0.599)	719 (0.593)	0.354/NS	0.918 (0.765–1.101)	0.806/NS	1.023 (0.852–1.228)
rs2795288	AA	80 (0.203)	91 (0.230)	140 (0.231)	0.297/NS	0.848 (0.622–1.156)	0.981/NS	0.996 (0.737–1.346)
	AT	213 (0.541)	190 (0.481)	325 (0.536)	0.894/NS	1.017 (0.789–1.312)	0.087/NS	0.801 (0.622–1.033)
	TT	101 (0.256)	114 (0.289)	141 (0.233)	0.393/NS	1.137 (0.847–1.526)	0.047/NS	1.338 (1.003–1.784)
	A	373 (0.473)	372 (0.471)	605 (0.499)	0.259/NS	0.902 (0.754–1.079)	0.216/NS	0.893 (0.746–1.068)
	T	415 (0.527)	418 (0.529)	607 (0.501)	0.259/NS	1.109 (0.927–1.327)	0.216/NS	1.120 (0.936–1.340)
rs844654	AA	121 (0.307)	110 (0.278)	182 (0.300)	0.820/NS	1.033 (0.784–1.360)	0.457/NS	0.899 (0.679–1.190)
	AT	208 (0.528)	211 (0.534)	311 (0.513)	0.649/NS	1.061 (0.823–1.367)	0.516/NS	1.088 (0.844–1.402)
	TT	65 (0.165)	74 (0.187)	113 (0.186)	0.385/NS	0.862 (0.616–1.206)	0.972/NS	1.006 (0.727–1.392)
	A	450 (0.571)	431 (0.546)	675 (0.557)	0.534/NS	1.059 (0.884–1.269)	0.617/NS	0.955 (0.798–1.144)
	T	338 (0.429)	359 (0.454)	537 (0.443)	0.534/NS	0.944 (0.788–1.131)	0.617/NS	1.047 ( (0.874–1.254)
rs12039904	CC	202 (0.513)	209 (0.529)	333 (0.550)	0.254/NS	0.836 (0.669–1.112)	0.527/NS	0.921 (0.714–1.188)
	CT	169 (0.429)	142 (0.259)	244 (0.403)	0.409/NS	1.114 (0.862–1.441)	0.170/NS	0.833 (0.641–1.082)
	TT	23 (0.058)	44 (0.111)	29 (0.048)	0.464/NS	1.233 (0.703–2.165)	1.58 × 10^−4^/4.74 × 10^−3^3	2.494 (1.532–4.060)
	C	573 (0.727)	560 (0.709)	910 (0.751)	0.237/NS	0.884 (0.721–1.084)	0.038/NS	0.808 (0.661–0.988)
	T	215 (0.273)	230 (0.291)	302 (0.249)	0.237/NS	1.131 (0.922–1.386)	0.038/NS	1.238 (1.012–1.513)
rs10912580	AA	230 (0.584)	234 (0.592)	329 (0.543)	0.204/NS	1.181 (0.914–1.526)	0.123/NS	1.224 (0.947–1.582)
	AG	129 (0.327)	133 (0.337)	237 (0.391)	0.041/NS	0.758 (0.581–0.989)	0.081/NS	0.790 (0.606–1.030)
	GG	35 (0.089)	28 (0.071)	40 (0.066)	0.181/NS	1.380 (0.860–2.213)	0.764/NS	1.080 (0.654–1.781)
	A	589 (0.747)	601 (0.761)	895 (0.738)	0.653/NS	1.048 (0.854–1.288)	0.262/NS	1.126 (0.915–1.386)
	G	199 (0.253)	189 (0.239)	317 (0.262)	0.653/NS	0.888 (0.721–1.093)	0.262/NS	0.954 (0.777–1.172)
rs844665	CC	276 (0.701)	294 (0.744)	423 (0.698)	0.933/NS	1.012 (0.767–1.334)	0.112/NS	1.259 (0.947–1.674)
	CT	110 (0.279)	98 (0.248)	165 (0.272)	0.811/NS	1.035 (0.780–1.375)	0.396/NS	0.882 (0.660–1.179)
	TT	8 (0.020)	3 (0.008)	18 (0.030)	0.361/NS	0.677 (0.292–1.572)	0.017/NS	0.250 (0.073–0.854)
	C	662 (0.840)	686 (0.868)	1011 (0.834)	0.725/NS	1.045 (0.819–1.332)	0.037/NS	1.311 (1.015–1.694)
	T	126 (0.160)	104 (0.132)	201 (0.166)	0.725/NS	0.957 (0.751–1.221)	0.037/NS	0.763 (0.590–0.985)
rs844644	AA	108 (0.274)	120 (0.304)	155 (0.256)	0.520/NS	1.099 (0.825–1.464)	0.096/NS	1.270 (0.958–1.683)
	AC	184 (0.467)	192 (0.486)	316 (0.521)	0.092/NS	0.804 (0.624–1.037)	0.274/NS	0.868 (0.674–1.119)
	CC	102 (0.259)	83 (0.210)	135 (0.223)	0.189/NS	1.219 (0.907–1.638)	0.636/NS	0.928 (0.682–1.264)
	A	400 (0.508)	432 (0.547)	626 (0.517)	0.698/NS	0.965 (0.807–1.155)	0.184/NS	1.130 (0.944–1.352)
	C	388 (0.492)	358 (0.453)	586 (0.483)	0.698/NS	1.036 (0.866–1.240)	0.184/NS	0.885 (0.740–1.060)

CI, confidence intervals; OR, odds ratios; NS, not significant; Pc value, the Bonferroni correction P values. Pc^a^ value, the Bonferroni correction P values for BD; Pc^b^ value the Bonferroni correction P values for VKH syndrome.

**Table 5:** Frequencies of alleles and genotypes of rs1234315 polymorphisms in BD patients and controls in second stage and combined results.

Genotype/allele	BD n (freq)	Controls n (freq)	P/Pc	OR (95% CI)	P^combined^/Pc^combined^	OR (95% CI)
CC	130 (0.239)	128 (0.310)	0.014/NS	0.699 (0.525–0.931)	3.80 × 10^−6^/1.14 × 10^−4^	0.615 (0.501–0.757)
CT	258 (0.474)	216 (0.523)	0.135/NS	0.823 (0.637–1.063)	0.861/NS	0.984 (0.824–1.175)
TT	156 (0.287)	69 (0.167)	1.53 × 10^−5^/4.59 × 10^−4^	2.004 (1.458–2.756)	4.80 × 10^−7^/1.44 × 10^−5^	1.734 (1.398–2.151)
C	518 (0.476)	472 (0.571)	3.57 × 10^−5^/1.07 × 10^−3^	0.682 (0.568–0.818)	5.33 × 10^−8^/1.60 × 10^−6^	0.688 (0.606–0.780)
T	570 (0.524)	354 (0.429)	3.57 × 10^−5^/1.07 × 10^−3^	1.467 (1.223–1.760)	5.33 × 10^−8^/1.60 × 10^−6^	1.455 (1.282–1.650)

**Table 6:** Frequencies of alleles and genotypes of rs12039904 polymorphisms in VKH patients and controls in second stage and combined results.

Genotype/allele	VKH n (freq)	Controls n (freq)	P/Pc	OR (95% CI)	P^combined^/Pc^combined^	OR (95% CI)
CC	463 (0.495)	666 (0.581)	8.13 × 10^−5^/2.44 × 10^−3^	0.705 (0.593–0.839)	3.11 × 10^−4^/9.33 × 10^−3^	0.769 (0.666–0.887)
CT	388 (0.415)	418 (0.365)	0.020/NS	1.233 (1.033–1.472)	0.251/NS	1.089 (0.941–1.261)
TT	85 (0.091)	62 (0.054)	1.14 × 10^−3^/0.034	1.746 (1.244–2.452)	1.54 × 10^−6^/4.62 × 10^−5^	1.959 (1.483–2.588)
C	1314 (0.702)	1750 (0.764)	7.29 × 10^−6^/2.19 × 10^−4^	0.729 (0.635–0.837)	1.16 × 10^−6^/3.48 × 10^−5^	0.755 (0.674–0.845)
T	558 (0.298)	542 (0.236)	7.29 × 10^−6^/2.19 × 10^−4^	1.371 (1.194–1.574)	1.16 × 10^−6^/3.48 × 10^−5^	1.325 (1.183–1.485)

## References

[b1] Yang P. . Clinical features of Chinese patients with Behcet’s disease. Ophthalmology 115, 312–318 e314 (2008). 17692378 10.1016/j.ophtha.2007.04.056

[b2] Yang P. . Clinical characteristics of Vogt–Koyanagi–Harada syndrome in Chinese patients. Ophthalmology 114, 606–614 (2007). 17123618 10.1016/j.ophtha.2006.07.040

[b3] Yang P. . Clinical patterns and characteristics of uveitis in a tertiary center for uveitis in China. Curr Eye Res 30, 943–948 (2005). 16282128 10.1080/02713680500263606

[b4] Bank I., Duvdevani M. & Livneh A. Expansion of gammadelta T-cells in Behcet’s disease: role of disease activity and microbial flora in oral ulcers. J Lab Clin Med 141, 33–40 (2003). 12518166 10.1067/mlc.2003.1

[b5] Chi W. . IL-23 promotes CD4+ T cells to produce IL-17 in Vogt-Koyanagi-Harada disease. J Allergy Clin Immunol 119, 1218–1224 (2007). 17335887 10.1016/j.jaci.2007.01.010

[b6] Sheu S. J. Update on uveomeningoencephalitides. Curr Opin Neurol 18, 323–329 (2005). 15891420 10.1097/01.wco.0000169753.31321.4e

[b7] Hou S. . Identification of a susceptibility locus in STAT4 for Behcet’s disease in Han Chinese in a genome-wide association study. Arthritis Rheum 64, 4104–4113 (2012). 23001997 10.1002/art.37708

[b8] Hou S. . Genome-wide association analysis of Vogt-Koyanagi-Harada syndrome identifies two new susceptibility loci at 1p31.2 and 10q21.3. Nat Genet 46, 1007–1011 (2014). 25108386 10.1038/ng.3061

[b9] Verity D. H., Marr J. E., Ohno S., Wallace G. R. & Stanford M. R. Behçet’s disease, the Silk Road and HLA-B51:historical and geographical perspectives. Tissue Antigens 54, 213–220 (1999). 10519357 10.1034/j.1399-0039.1999.540301.x

[b10] Remmers E. F. . Genome-wide association study identifies variants in the MHC class I, IL10, and IL23R-IL12RB2 regions associated with Behçet’s disease. Nat Genet 42, 698–702 (2010). 20622878 10.1038/ng.625 PMC2923807

[b11] Mizuki N. . Genome-wide association studies identify IL23R-IL12RB2 and IL10 as Behçet’s disease susceptibility loci. Nat Genet 42, 703–706 (2010). 20622879 10.1038/ng.624

[b12] Zhao M., Jiang Y. & Abrahams I. W. Association of HLA antigens with Vogt-Koyanagi-Harada syndrome in a Han Chinese population. Arch Ophthalmol 109, 368–370 (1991). 2003797 10.1001/archopht.1991.01080030070041

[b13] Shu Q. . Interleukin-17 gene polymorphism is associated with Vogt-Koyanagi-Harada syndrome but not with Behçet’s disease in a Chinese Han population. Hum Immunol 71, 988–991 (2010). 20620187 10.1016/j.humimm.2010.06.020

[b14] Bossini-Castillo L. . A replication study confirms the association of TNFSF4 (OX40L) polymorphisms with systemic sclerosis in a large European cohort. Ann Rheum Dis 70 **(4)**, 638–641 (2011). 21187296 10.1136/ard.2010.141838

[b15] Delgado-Vega A. . Replication of the TNFSF4 (OX40L) promoter region association with systemic lupus erythematosus. Genes & Immunity 10 **(3)**, 248–253 (2009). 19092840 10.1038/gene.2008.95 PMC3867640

[b16] Chang Y. K. . Association of BANK1 and TNFSF4 with systemic lupus erythematosus in Hong Kong Chinese. Genes Immun 10 **(5**), 414–420 (2009). 19357697 10.1038/gene.2009.16 PMC2834352

[b17] Zhou X. J. . Gene-gene interaction of BLK, TNFSF4, TRAF1, TNFAIP3, and REL in systemic lupus erythematosus. Arthritis Rheum 64 **(1)**, 222–231 (2012). 21905002 10.1002/art.33318 PMC3994469

[b18] Zhang S. Q. . A single-nucleotide polymorphism of the TNFSF4 gene is associated with systemic lupus erythematosus in Chinese Han population. Rheumatol Int 31 **(2)**, 227–231 (2011). 20012871 10.1007/s00296-009-1247-2

[b19] Lu M. M. . Association of TNFSF4 polymorphisms with systemic lupus erythematosus: a meta-analysis. Mod Rheumatol 23 **(4)**, 686–693 (2013). 22850862 10.1007/s10165-012-0708-8

[b20] Lee Y. H. & Song G. G. Associations between TNFSF4 and TRAF1-C5 gene polymorphisms and systemic lupus erythematosus: a meta-analysis. Hum Immunol 73 **(10)**, 1050–1054 (2012). 22820624 10.1016/j.humimm.2012.07.044

[b21] Gourh P. . Association of TNFSF4 (OX40L) polymorphisms with susceptibility to systemic sclerosis. Ann Rheum Dis 69 **(3)**, 550–555 (2010). 19778912 10.1136/ard.2009.116434 PMC2927683

[b22] Nordmark G. . Association of EBF1, FAM167A(C8orf13)-BLK and TNFSF4 gene variants with primary Sjögren’s syndrome. Genes Immun 12 **(2)**, 100–109 (2010). 20861858 10.1038/gene.2010.44

[b23] Croft M. The role of TNF superfamily members in T-cell function and diseases. Nat Rev Immunol 9, 271–285 (2009). 19319144 10.1038/nri2526 PMC2737409

[b24] Baum P. R. . Molecular characterization of murine and human OX40/OX40 ligand systems: identification of a human OX40 ligand as the HTLV-1-regulated protein gp34. E MBO J 13, 3992–4001 (1994). 10.1002/j.1460-2075.1994.tb06715.x PMC395319 8076595

[b25] Latza U. . The human OX40 homolog: cDNA structure, expression and chromosomal assignment of the ACT35 antigen. Eur J Immunol 24, 677–683 (1994). 7510240 10.1002/eji.1830240329

[b26] Ito T. . OX40 ligand shuts down IL-10-producing regulatory T cells. Proc Natl Acad Sci USA 03, 13138–13143 (2006). 10.1073/pnas.0603107103 PMC1559766 16924108

[b27] Li J. . Negative regulation of IL-17 production by OX40/OX40L interaction. Cell Immunol 253, 31–37 (2008). 18501882 10.1016/j.cellimm.2008.04.010

[b28] Gramaglia I., Weinberg A. D., Lemon M. & Croft M. Ox-40 ligand: a potent costimulatory molecule for sustaining primary CD4 T cell responses. J Immunol 161, 6510–6517 (1998). 9862675

[b29] XiaoX. . OX40/OX40L costimulation affects induction of Foxp3+ regulatory T-cells in part by expanding memory T-cells *in vivo*. J Immunol 181, 3193–3201 (2008).1871399010.4049/jimmunol.181.5.3193

[b30] Zhou Y. B., Ye R. G., Li Y. J. & Xie C. M. Targeting the CD134-CD134L interaction using anti-CD134 and/or rhCD134 fusion protein as a possible strategy to prevent lupus nephritis. Rheumatology International 29 **(4)**, 417–425 (2009). 18802705 10.1007/s00296-008-0697-2

[b31] Farres M. N., Al-Zifzaf D. S., Aly A. A. & Abd Raboh N. M. OX40/OX40L in systemic lupus erythematosus: association with disease activity and lupus nephritis. Annals of Saudi Medicine 31 **(1)**, 29–34 (2011). 21245596 10.4103/0256-4947.75775 PMC3101721

[b32] Wu X. . Activation of OX40 Prolongs and Exacerbates Autoimmune Experimental Uveitis. Investigative Ophthalmology & Visual Science 52 **(11)**, 8520–8526 (2011). 21948545 10.1167/iovs.11-7664 PMC3208192

[b33] Manku H., Graham D. S. & Vyse T. J. Association of the co-stimulator OX40L with systemic lupus erythematosus. J Mol Med 87 **(3)**, 229–234 (2009). 19083191 10.1007/s00109-008-0431-2

[b34] Fresko I. . Genetic anticipation in Behcet’s syndrome. Ann Rheum Dis 57, 45–48 (1998). 9536823 10.1136/ard.57.1.45 PMC1752455

[b35] Ohno S. Immunological aspects of Behcet’s and Vogt–Koyanagi-Harada’s diseases. Trans Ophthalmol Soc K 10, 335–341 (1981). 6820735

[b36] Yamaki K., Gocho K. & Sakuragi S. Pathogenesis of Vogt-Koyanagi-Harada disease. Int Ophthalmol Clin 42, 13–23 (2002). 10.1097/00004397-200201000-00004 12189608

[b37] Ishikawa A., Shiono T. & Uchida S. Vogt–Koyanagi–Harada disease in identical twins. Retina 14, 435–437 (1994). 7899719 10.1097/00006982-199414050-00008

[b38] Chen F. . CD40 gene polymorphisms confer risk of Behcet’s disease but not of Vogt-Koyanagi-Harada syndrome in a Han Chinese population. Rheumatology (Oxford) 51, 47–51 (2012). 22087016 10.1093/rheumatology/ker345

[b39] Hou S. . Genome-wide association study identifies susceptible locus in STAT4 for Behcet’s disease in Han Chinese. Arthritis Rheum 64 **(12)**, 4104–4013 (2012). 23001997 10.1002/art.37708

[b40] Hu K., Hou S., Jiang Z., Kijlstra A. & Yang P. JAK2 and STAT3 polymorphisms in a Han Chinese population with Behcet’s disease. Invest Ophthalmol Vis Sci 53, 538–541 (2012). 22205606 10.1167/iovs.11-8440

[b41] Hu K. . STAT4 polymorphism in a Chinese Han population with Vogt–Koyanagi–Harada syndrome and Behcet’sdisease. Human Immunology 71, 723–726 (2010). 20438790 10.1016/j.humimm.2010.04.007

[b42] Sugita S. . Ocular infiltrating CD4+ T cells from patients with Vogt-Koyanagi-Harada disease recognize human melanocyte antigens. Invest Ophthalmol Vis Sci 47, 2547–2554 (2006). 16723469 10.1167/iovs.05-1547

[b43] Ohno S. Immunological aspects of Behcet’s and Vogt-Koyanagi-Harada’s diseases. Trans Ophthalmol Soc UK 101, 335–341 (1981). 6820735

[b44] Criteria for diagnosis of Behcet’s disease. International Study Group for Behcet’s disease. Lancet 335, 1078–1080 (1990). 1970380

[b45] Read R. W. . Revised diagnostic criteria for Vogt–Koyanagi–Harada disease: Report of an international committee on nomenclature. Am J Ophthalmol 131, 647–652 (2001). 11336942 10.1016/s0002-9394(01)00925-4

